# PITPNA-AS1/miR-98-5p to Mediate the Cisplatin Resistance of Gastric Cancer

**DOI:** 10.1155/2022/7981711

**Published:** 2022-05-07

**Authors:** Zhongling Ma, Gang Liu, Suhong Hao, Tianfu Zhao, Wei Chang, Jiansheng Wang, Hong Ren, Xinhan Zhao

**Affiliations:** ^1^Department of Oncology, The First Affiliated Hospital of Xi'an Jiaotong University, 277 West Yanta Road, Xi'an, 710061 Shaanxi Province, China; ^2^Department of Oncology, Xi'an Chang'an District Hospital, No. 120 Wenyuan Middle Road, Chang'an District, Xi'an, 710100 Shaanxi Province, China; ^3^Department of Surgical Thoracic, The First Affiliated Hospital of Xi'an Jiaotong University, 277 West Yanta Road, Xi'an, 710061 Shaanxi Province, China

## Abstract

Gastric cancer (GC) is the most deadly gastrointestinal malignancy with high incidence and mortality. Although, molecular mechanisms which drive gastric cancer progression are extensively investigated, the roles of long noncoding RNA (lncRNA) in gastric cancer growth and drug sensitivity remain unclear. Platinum is a mainstay to treat gastric cancer, and platinum resistance always leads to the local recurrence of gastric cancer. Therefore, it is important to identify biomarkers or therapeutic targets to sensitize gastric cancer to platinum. In this study, we employ noncoding RNA sequencing and found that lncRNA PITPNA-AS1 is overexpressed in gastric cancer tissues and associated with poor survival of gastric cancer patients. Kockdown of PITPNA-AS1 in gastric cancer cells significantly inhibited cell growth and triggered apoptotic cell death in gastric cancer cells. Also, cisplatin treatment could decrease PITPNA-AS1 levels in gastric cancer cells through inhibiting H3K27ac. Besides, PITPNA-AS1 is elevated in cisplatin-resistant gastric cancer cells and tissues, PITPNA-AS1 knockdown could sensitize gastric cancer cells to cisplatin treatment. Furthermore, we identified that PITPNA-AS1 directly interacts and inhibits miR-98-5p. Therefore, PITPNA-AS1 could be served as a potential biomarkers and curative therapeutic targets for gastric cancer progression.

## 1. Introduction

Gastric cancer (GC) is the fourth most commonly diagnosed cancer and the second most common cancer-related mortality globally, with approximately 738,000 people died of GC each year worldwide [1, 2]. Despite the remarkable progressive improvement in surgical and medical techniques, prognosis of patients with GC remains relatively poor, mainly due to its high recurrence and metastasis incidence [3]. Neoadjuvant chemotherapy improves overall survival of GC patients in comparison to traditional chemotherapy or surgery alone. 5-Fluorouracil combined with cisplatin has been convincingly proved survival benefits for HER-2-positive patients [4–6]. As the main chemotherapy treatment for postoperative GC patients, the efficacy of platinum has been largely limited due to the chemo-resistance [7]. Laboratory studies illustrated that resistance to platinum is almost multifactorial, which includes impaired cellular uptake of platinum drug [8], reinforced endocellular detoxification by glutathione and metallothionein systems [9], enhanced DNA repair capacity, enhanced tolerance to DNA damage [10], and rising restore of DNA damage [8, 11].

Long noncoding RNAs (LncRNAs) are a class of noncoding RNAs longer than 200 nt without protein coding potential. Several lncRNAs were confirmed as biotargets for modulating cisplatin resistance in cancer through the cell cycle, apoptosis, and Wnt pathways [12], which acts as a competing endogenous RNA or directly binding to mRNAs or proteins and regulating their expression and functions [13]. LncRNA PITPNA antisense RNA 1 (PITPNA-AS1) is located in chromosome 17p13.3, with the function of regulating cell growth and motility of hepatocellular carcinoma via miR-876-5p/WNT5A pathway, which was affirmed by rescue and in vivo experiments [14]. Furthermore, PITPNA-AS1 was found to be involved in promoting EMT process to promote proliferation and metastasis of non-small-cell lung cancer. Based on the fact that EMT is an important mechanism for regulating platinum resistance, we hypothesized that PITPNA-AS1 took part in mediating platinum resistance as well.

MiRNAs are another type of noncoding RNAs with 19-24 nt in length, which could posttranscriptionally repress gene expression via binding to the 3′-untranslated region (3′-UTR) of mRNA [15]. Wei et al. summarized expression levels and potential targets of 53 microRNAs (miRNAs) which participated in platinum resistance of gastric cancer [16]. It has been reported that several oncogenic miRNAs can promote platinum resistance of gastric cancer, such as miR-20a [17] and miR-106a [18], while tumor suppressive miRNAs can reverse platinum resistance, such as miR-508p [19] and miR-129-5p [20]. As a valid tumor suppressor, microRNA-98-5p (miR-98-5p), which is one member of let-7 family, is usually downregulated in various cancers, such as nasopharyngeal carcinoma [21] and endometrial cancer [22]. But increased expression of miR-98-5p has been observed in primary breast cancer swatches [23]. Perhaps miR-98 has completely opposite obligation in different types of cancers. A series of assays have elucidated that MiR-98-5p was expressed significantly lower in pancreatic ductal adenocarcinoma tissues compared with normal tissue and its expression was highly associated with tumor size, TNM stage, lymph node metastasis, and survival. And it could negatively regulate MAP4K4 and inhibit MAPK/ERK signaling [24]. Until now, few studies implemented the function of miR-98-5p in GC.

Although recent advanced studies identify molecular elements of GC, the precise mechanisms of tumourigenesis remain largely unknown [25]. Therefore, the clarification of new pathogenesis is vital for practical targeted treatment for GC; many studies verified that lncRNA and miRNA played vital functions in the development and therapeutic resistance of cancers and their aberrant expression emerged as important hallmarks of multiple cancers [26–28]. However, few studies reported the molecular mechanisms of PITPNA-AS1 and miR-98-5p in GC, especially when it comes to their relationship with platinum resistance. Thus, we investigate the role of PITPNA-AS1 and miR-98-5p in GC and their connection with platinum resistance.

## 2. Results

### 2.1. RNA Sequencing for lncRNA and MicroRNA in 3 Gastric Cancer Patients

**Figure 1:**
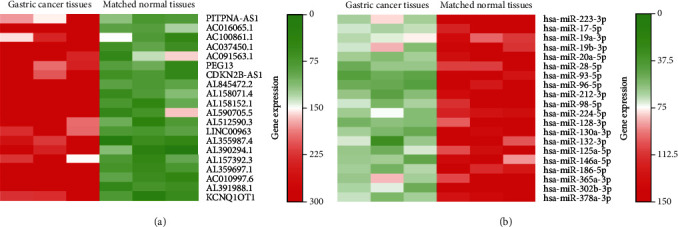
RNA sequencing for lncRNA and microRNA in 3 gastric cancer patients. (a) Heatmap based on the lncRNA NGS between gastric cancer and matched normal tissues to show the top 20 differentially expressed genes. (b) Heatmap based on the microRNA NGS between gastric cancer and matched normal tissues to show the top 20 differentially expressed microRNAs in gastric cancer.

To identify the differentiated expression noncoding RNA (ncRNA) in cisplatin sensitive and cisplatin resistant gastric cancer tissues, we have performed ncRNA sequencing including lncRNA and microRNA using gastric cancer tissue (cisplatin sensitive vs. resistant). The top 20 upregulated lncRNAs and microRNAs including PITPNA-AS1 are shown in Figure 1. Elevated expression of PITPNA-AS1 was previously detected in gastric cancers; our ncRNA sequencing further showed that PITPNA-AS1 was downregulated in cisplatin resistant gastric cancer. To identify the downstream effector of PITPNA-AS1, we used DIANA on-line software, which is a website-based tool to prediction miRNA-lncRNA interactions, and found that miR-98-5p might be the target of PITPNA-AS1.

### 2.2. PITPNA-AS1 Expression Was Correlated with Local Recurrence in Gastric Cancer Patients

**Figure 2:**
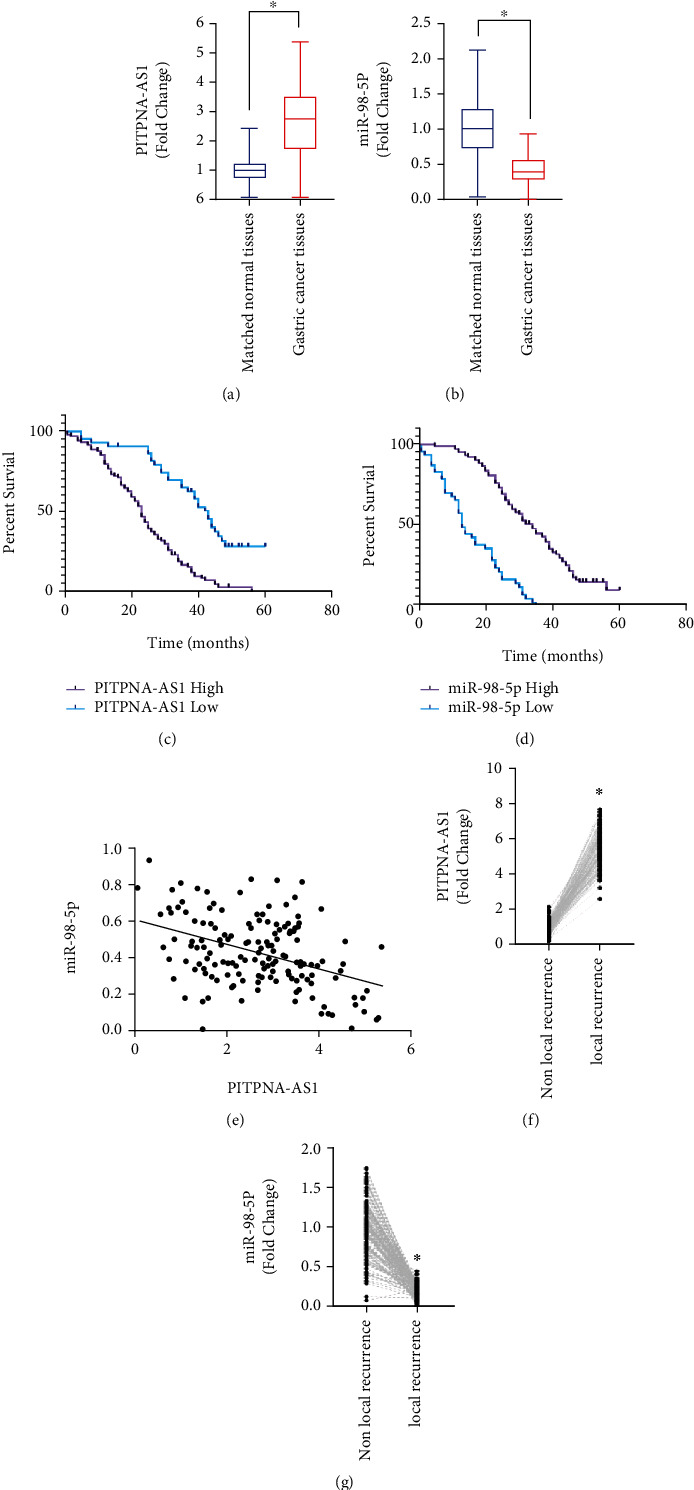
PITPNA-AS1 expression was correlated with local recurrence in gastric cancer patients. (a) PITPNA-AS1 expression was measured using qRT-PCR method in gastric cancer tissues compared with matched normal tissues. (b) miR-98-5p expression was measured using qRT-PCR method in gastric cancer tissues compared with matched normal tissues. (c) Survival analysis shown as KM-plot for PITPNA-AS1 high-expression group and low-expression group in gastric cancer patients. (d) Survival analysis shown as KM-plot for miR-98-5p high-expression group and low-expression group in gastric cancer patients. (e) The correlation of PITPNA-AS1 and miR-98-5p based on qRT-PCR method in gastric cancer patients. (f) PITPNA-AS1 expression was measured by qRT-PCR in gastric cancer patients stratified by local recurrence and nonlocal recurrence. (g) miR-98-5p expression was measured by qRT-PCR in gastric cancer patients stratified by local recurrence and nonlocal recurrence.

To further confirm the PITPNA-AS1 expression in gastric cancer tissues compared with their matched normal tissue, we have measured PITPNA-AS1 expression in 153 gastric cancer tissues compared with para-cancer tissues and found that PITPNA-AS1 was significantly increased cancer tissues; meanwhile, the level of its putative target miR-98-5p significantly downregulated in cancer tissues (Figures 2(a) and 2(b)). In addition, the patients' survival analysis showed that the high expression of PITPNA-AS1 was associated with poor survival (Figure 2(c)). On the contrary, the high level of miR-98-5p was associated with better overall survival (Figure 2(d)). Besides, the expression of PITPNA-AS1 was negatively correlated with miR-98-5p expression in gastric cancer tissues (Figure 2(e)). Furthermore, significant higher level of PITPNA-AS1 has been detected in local recurrent gastric cancer patients compared with nonrecurrent cancer tissues, while miR-98-5p was downregulated in local recurrent gastric cancer tissues (Figures 2(f) and 2(g)).

### 2.3. PITPNA-AS1/miR-98-5p Regulated Cell Proliferation and Inhibits Apoptosis in Gastric Cancer Cell Lines

**Figure 3:**
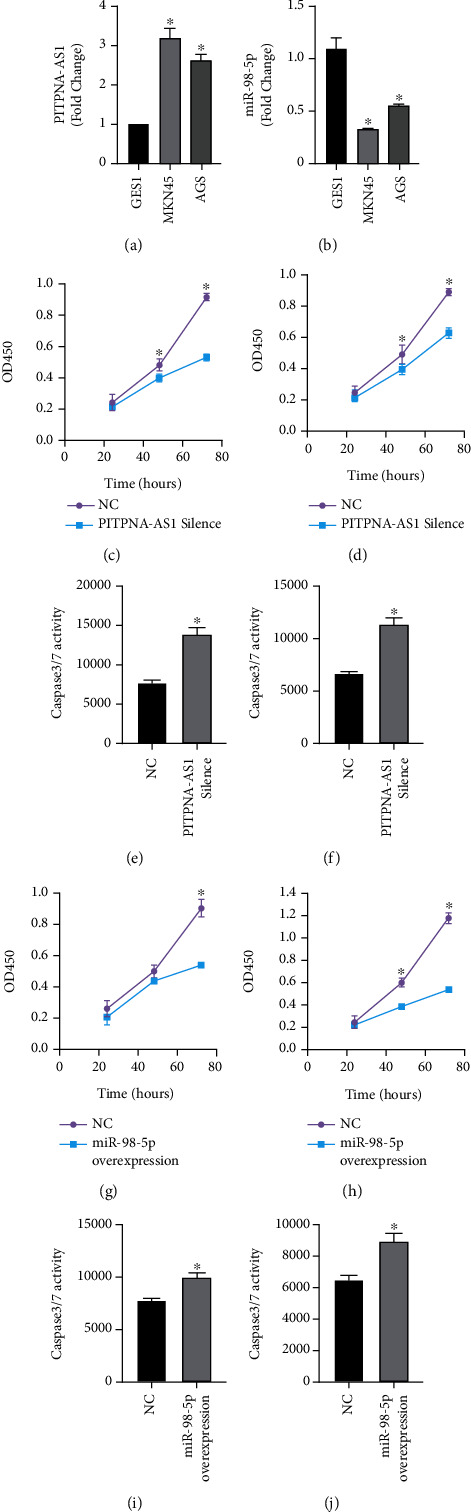
PITPNA-AS1/miR-98-5p regulated cell proliferation and inhibits apoptosis in gastric cancer cell lines. (a) PITPNA-AS1 expression was measured by qRT-PCR in gastric cancer cells, MKN45 and AGS compared with normal gastric cell GES1. (b) miR-98-5p expression was measured by qRT-PCR in gastric cancer cells, MKN45 and AGS compared with normal gastric cell GES1. (c) Cell viability was measured by CCK-8 assay for PITPNA-AS1 silence AGS cells and negative control plasmids transduced AGS cells. (d) Cell viability was measured by CCK-8 assay for PITPNA-AS1 silence MKN45 cells and negative control plasmids transduced MKN45 cells. (e) Apoptosis was measured by caspase 3/7 activity kit for PITPNA-AS1 silence AGS cells and negative control plasmids transduced AGS cells. (f) Apoptosis was measured by caspase 3/7 activity kit for PITPNA-AS1 silence MKN45 cell line and negative control plasmids transduced MKN45 cells. (g) Cell viability was measured by CCK-8 assay for miR-98-5p overexpression AGS cells and negative control plasmids transduced AGS cells. (h) Cell viability was measured by CCK-8 assay for miR-98-5p overexpression MKN45 cells and negative control plasmids transduced MKN45 cells. (i) Apoptosis was measured by caspase 3/7 activity kit for miR-98-5p overexpression AGS cells and negative control plasmids transduced AGS cells. (j) Apoptosis was measured by caspase 3/7 activity kit for miR-98-5p overexpression MKN45 cells and negative control plasmids transduced MKN45 cells.

Next, we measured the PITPNA-AS1 levels in gastric cancer cells, and found that PITPNA-AS1 was overexpressed in human gastric cancer cell lines including MKN45 and AGS, but not in in normal gastric mucosal cell line GES-1 (Figure 3(a) and Sup Figure 1). Meanwhile the expression of miR-98-5p was lower in MKN45 and AGS than that in GSE-1 cell line (Figure 3(b)). To investigate the biological roles of PITPNA-AS1 in gastric cancer, knocked down the expression of PITPNA-AS1 in MKN45 and AGS cells (Sup Figures 2a and 2b), and found that silence of PITPNA-AS1 significantly inhibited cancer cell proliferation (Figures 3(c) and 3(d)). Also, PITPNA-AS1 knockdown caused apoptotic cell death in MKN45 and AGS cells, as evidenced by significant increased caspase 3/7 activity. In contrast with PITPNA-AS1, ectopic expression of miR-98-5p (Sup Figures 2c and 2d) could significantly decreased cell proliferation rate and enhanced cell apoptosis rate (Figures 3(g)–3(j)).

### 2.4. PITPNA-AS1 Negatively Regulated the Expression of miR-98-5p

**Figure 4:**
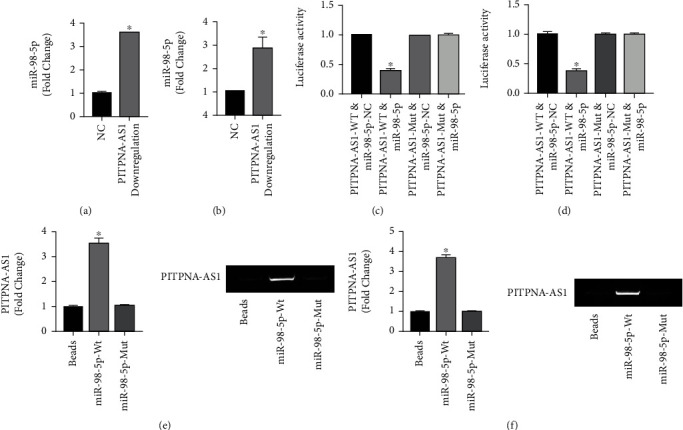
PITPNA-AS1 negatively regulated the expression of miR-98-5p. (a) miR-98-5p expression was measured by qRT-PCR in PITPNA-AS1 silence AGS cells. (b) miR-98-5p expression was measured by qRT-PCR in PITPNA-AS1 silence MKN45 cells. (c) Dual-luciferase reporter gene assay was used to investigate the interaction between miR-98-5p and PITPNA-AS1 in AGS cell line. (d) Dual-luciferase reporter gene assay was used to investigate the interaction between miR-98-5p and PITPNA-AS1 in MKN45 cell line. (e) RNA pull down assay following qRT-PCR and PCR agarose gel electrophoresis was used to investigate the interaction between miR-98-5p and PITPNA-AS1 in AGS cell line. (f) RNA pull down following qRT-PCR and PCR agarose gel electrophoresis was used to investigate the interaction between miR-98-5p and PITPNA-AS1 in MKN45 cell line.

To validate whether miR-98-5p could be the target of PITPNA-AS1, we examined the miR-98-5p expression after PITPNA-AS1 knockdown and found that silence of PITPNA-AS1 significantly increased the level of miR-98-5p (Figures 4(a) and 4(b)). Then, we performed dual luciferase reporter gene assay and found that PITPNA-AS1 was associated with miR-98-5p in cell (Figures 4(c) and 4(d)). We further performed *in vitro* RNA pulldown assay and found that PITPNA-AS1 directly interacted with miR-98-5p (Figures 4(e) and 4(f)).

### 2.5. PITPNA-AS1 Expression Can Be Suppressed by Cisplatin in Gastric Cancer Cell Lines

**Figure 5:**
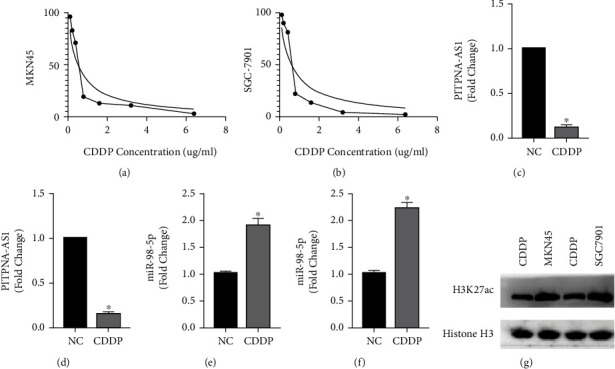
PITPNA-AS1 expression can be suppressed by cisplatin in gastric cancer cell lines. (a) IC50 of AGS to CDDP was detected by CCK-8 assay. (b) IC50 of MKN45 to CDDP was detected by CCK-8 assay. (c) CDDP suppressed PITPNA-AS1 expression, which was measured by qRT-PCR, in AGS cell line. (d) CDDP, which was measured by qRT-PCR, suppressed PITPNA-AS1 expression in MKN45 cell line. (e) CDDP induced miR-98-5p expression, which was measured by qRT-PCR, in AGS cell line. (f) CDDP induced miR-98-5p expression, which was measured by qRT-PCR, in MKN45 cell line. (g) CDDP suppressed H3K27ac expression, which was measured by Western blot, in MKN45 and AGS cell lines.

PITPNA-AS1 was decreased in the cisplatin-resistant gastric cancer tissues; we then examined whether cisplatin treatment whether could affect PITPNA-AS1 expression. As shown in Figure 5(a), the half maximal inhibitory concentration (IC50) of CDDP in MKN45 is 0.52*μ*g/mL and the IC50 in AGS is 0.59 *μ*g/mL (Figure 5(b)). We used CDDP (0.52 *μ*g/mL) to treat MKN45 and CDDP (0.59 *μ*g/mL) to treat AGS cells for 24 hours and found that PITPNA-AS1 expression can be significantly suppressed after cisplatin treatment (Figures 5(c) and 5(d)).

In the meantime, we also detected that CDDP treatment could significantly increase expression of miR-98-5p in MKN45 (0.52 *μ*g/mL) and in AGS cells (0.59 *μ*g/mL) (Figures 5(e) and 5(f)). To study the mechanism which leads to PITPNA-AS1 downregulation during cisplatin treatment, we examined H3K27ac levels after cisplatin treatment, since H3K27ac is a well-established marker for active enhancers and promoters. As shown in Figure 5(g), we found that H3K27ac expression was significantly suppressed in MKN45 and AGS cells when treated with CDDP.

### 2.6. PITPNA-AS1/miR-98-5p Regulated by H3K27ac Influenced the Effect of Platinum

**Figure 6:**
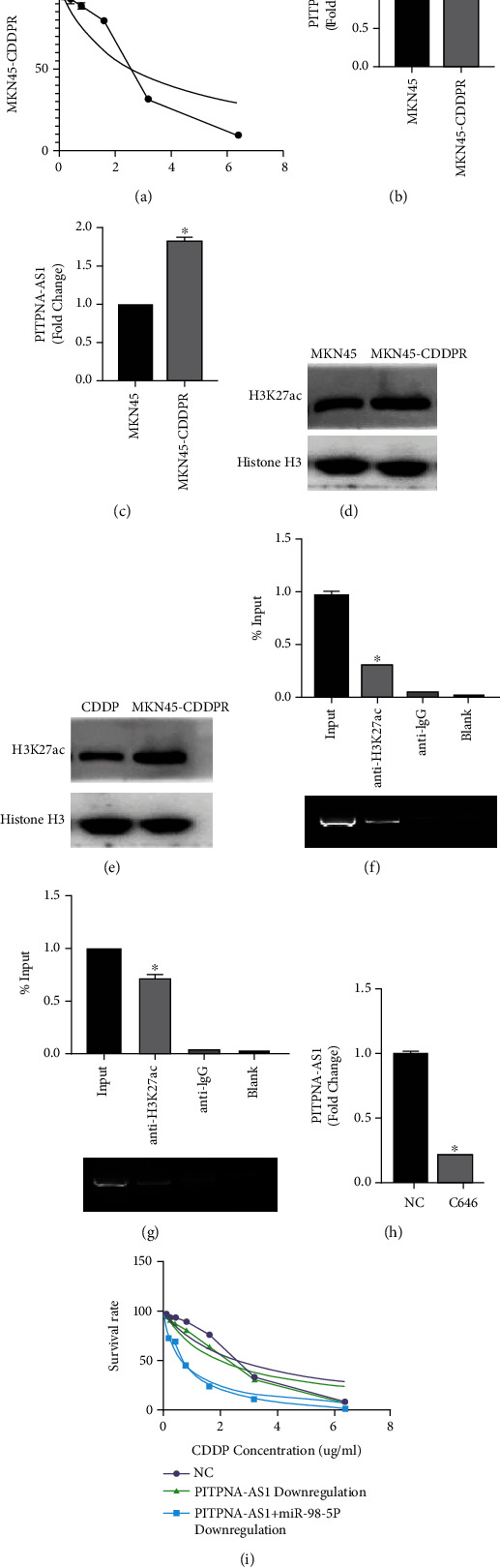
PITPNA-AS1/miR-98-5p regulated by H3K27ac influenced the effect of platinum. (a) IC50 of MKN45-CDDPR to CDDP was detected by CCK-8 assay. (b) PITPNA-AS1 expression, which was measured by qRT-PCR, in MKN45-CDDPR cell line was detected by CCK-8 assay. (c) miR-98-5p expression, which was measured by qRT-PCR, in MKN45-CDDPR cell line. (d) H3K27ac expression, which was measured by Western blot, in MKN45-CDDPR cell line. (e) CDDP suppressed the H3K27ac expression, which was measured by Western blot, in MKN45-CDDPR cell line. (f) RIP assay was performed to show that H3K27ac enriched in the promotor region of PITPNA-AS1 in parental cells. (g) RIP assay was performed to show that H3K27ac enriched more in the promotor region of PITPNA-AS1 in MKN45-CDDPR cell line. (h) C646 suppressed the PITPNA-AS1 expression, which was measured by qRT-PCR. (i) CCK-8 assay showed that PITPNA-AS1 knock down suppressed IC50 of MKN45-CDDPR, which be reversed by miR-98-5p knock down.

We have generated cisplatin-resistant MKN45 cells (MKN45-CDDPR), the IC50 of which is 2.60 *μ*g/mL, which MKN45 parental cell has a IC50 of 0.59*μ*g/mL (Figure 6(a)). Then, we checked PITPNA-AS1 expression in MKN45 parental and cisplatin resistant cells, and we found that PITPNA-AS1 was overexpressed in MKN45-CDDPR compared with parental cells (Figure 6(b)); meanwhile, we also detected that miR-98-5p was downregulated in cisplatin-resistant cells (Figure 6(c)).

Furthermore, we also found that H3K27ac was upregulated in MKN45-CDDPR cells (Figure 6(d)), which could be significantly suppressed by cisplatin treatment (Figure 6(e)). Furthermore, Chip assay showed that H3K27ac enriched more in the promotor region of PITPNA-AS1 in MKN45-CDDPR cells than in parental cells (Figures 6(f) and 6(g)). By treating with C646, the expression of PITPNA-AS1 in MKN45-CDDPR could be significantly suppressed (Figure 6(h)). Then, we transfected PITPNA-AS1-WT plasmids and found that PITPNA-AS1 knock down could suppress IC50 of MKN45-CDDPR, which could be reversed by miR-98-5p knock down (Figure 6(i)).

## 3. Discussion

Collectively, in this study, we discovered the role of PITPNA-AS1 and miR-98-5p in gastric cancer through gain and loss-of-function assays and analyzed the mechanism by which PITPNA-AS1 regulates apoptosis and drug resistance through the miR-98-5p targeting axis.

Gastric cancer is one of the leading public health problems worldwide because of its high incidence, morbidity, and mortality rate [29]. Currently, lacking of screening methods and early symptom, patients are most often diagnosed at advanced stages, with metastatic at distant sites and somber prognosis (median overall survival is 10-12 months) [30, 31]. For locally advanced disease, adjuvant or neoadjuvant therapy which recognized as the optimal therapeutic option is usually introduced with surgery owing to its curability [30]. Fluoropyrimidine plus oxaliplatin doublet is considered as the preferred first-line regimen due to its comparable survival benefits and lower toxicity [32]. Overcoming resistance is still a challenge in GC chemotherapy.

LncRNAs are associated with the tumor recurrence and poor prognosis, and abnormal expression has been observed in various tumors [33]. Mounting evidence elucidated that lncRNAs could act as oncogenes or tumor suppressors by modulating the gene expression or function in tumorigenesis [34], which possibly induce significant influence on the alterations of cell proliferation, metastasis, autophagy, and apoptosis [35, 36]. Our study indicated that lncRNA PITPNA-AS1 was highly expressed in gastric cancer patients and was associated with poor prognosis. Alteration of gene expression is correlated with the cancer specific survival of patients. PITPNA-AS1 was overexpressed in MKN45 cell line while knocking down PITPNA-AS1 resulted in inhibiting cell proliferation rate and increasing apoptosis rate. We first time inspected the role of PITPNA-AS1 in GC, which founding the basis for further exploration.

Next, we investigated the potential mechanism underlying PITPNA-AS1. Biased on current study, mechanism assays unveiled that PITPNA-AS1 targeted miR-98-5p. Dual-luciferase reporter gene assay, RNA pull-down assay, and RIP consequence provided powerful evidence that PITPNA-AS1 could interact with miR-98-5p. Moreover, knocking down of PITPNA-AS1 resulted in decreased expression of miR-98-5p, which confirmed this discovery again. The antitumor function of miR-98-5p has been documented yet. For instances, Fu et al. recognized miR-98-5p underexpression as biomarkers for predicting poor prognosis in pancreatic ductal adenocarcinoma (PDAC) patients because miR-98-5p inhibits proliferation and metastasis via targeting MAP4K4 [24]. Acting as a tumor suppressor, miR-98 could decelerate cancer aggressiveness by inhibiting TWIST expression in non-small-cell lung cancers [37]. In hepatocellular carcinoma (HCC), miR-98-5p could restrain cell proliferation and induce cell apoptosis via inhibition of its target gene IGF2BP1 [38]. As for colon cancer, miR-98 plays the role of tumor suppressor gene and inhibits Warburg effect by targeting HK2 (HK2 involves in miR-98-mediated suppression of glucose uptake, lactate production, and cell proliferation, whose expression was negatively correlated with miR-98) in colon cancer cells, which provided promising therapeutic candidate for clinical treatments [39].

In our study, miR-98-5p was shown to be downregulated in GC. Overexpression of miR-98-5p led to decreased cell proliferation rate and ascended apoptosis rate. Moreover, inhibition of miR-98-5p partially reversed the inhibitory effects of PITPNA-AS1 on GC cell proliferation and apoptosis. Thus, we draw the conclusion that PITPNA-AS1 exerts its tumor-promotion effect in GC via negatively modulating the expression of miR-98-5p. Laboratory findings were consistent with literature reports. Guo et al. revealed that lncRNA PITPNA-AS facilitates the cervical cancer progression on the proliferation, cell cycle, and apoptosis by targeting the miR-876-5p/c-MET axis [40].

It is well-established that aberrant lncRNA expression is strongly implicated in drug resistance in some cancers [41, 42]. Our experiments uncovered that cisplatin (CDDP) and lobaplatin (LBP) could suppress PITPNA-AS1 expression and induce expression of miR-98-5p in GC cell lines. Besides, PITPNA-AS1 was overexpressed in MKN45-CDDPR and MKN45-LBPR, which could confer GC cell resistance to platinum drugs, compared with their parental cells. However, miR-98-5p has the opposite effects. Furthermore, PITPNA-AS1-WT could reverse the inhibitory effect of platinum. These data demonstrated that PITPNA-AS1/miR-98-5p had a major role in regulating platinum-resistant in GC cells. Consistent with aforementioned findings, Wang's studies have identified that elevated expression of miR-98-5p is associated with resistance to cisplatin treatment through directly targeting Dicer1 and poor clinical outcomes in epithelial ovarian cancer patients [43]. Guo's studies have illustrated that cancer-associated fibroblast- derived exosomal who carrying overexpressed miR-98-5p promoted cisplatin resistance in ovarian cancer by downregulating CDKN1A [44]. An existing study has revealed that miR-129 could enhance chemosensitivity to cisplatin by suppressing P-gp protein in GC cells [45].

Conjointly, these results suggest that miR-98-5p could be served as a novel prognostic factors and critical therapeutic target in GC by enhancing chemo-sensitivity for platinum treatment against GC. However, downstream signal molecule and other biological processes are required further investigation.

## 4. Materials and Methods

### 4.1. Gastric Cancer Patients

The GC tissues and local recurrence GC tissues, as well as the corresponding para-cancer tissues, were collected from the patients who were diagnosed as GC by surgical resection at the First Affiliated Hospital of Xi'an Jiao Tong University. All patients were treated with 800 mg/m^2^ fluorouracil (civ 24 h, d1~5) and 80 mg/m^2^ cisplatin (ivgtt, d1) for 2 cycles before surgery and 2-4 cycles after surgery. Routine blood test and chest and abdominal CT were performed every 2 months during the follow-up. Local recurrence was determined based on the CT results. All of the samples were pathologically diagnosed and stored in liquid nitrogen. All of the patients had signed a written informed consent. The present study gained approval from the Ethics Committee of The First Affiliated Hospital of Xi'an Jiao tong University. And all experiments were conducted in accordance with relevant guidelines and regulations, which is consistent with the Declaration of Helsinki regulations.

### 4.2. Cell Culture

The human GC cell lines MKN45 and AGS were purchased from Shanghai Gaining Biological Technology Co., Ltd. (Shanghai, China), and the human gastric epithelial cell line GES-1 was obtained from American Type Culture Collection (Virginia, USA). All the cells were cultured in DMEM medium (HyClone, USA) containing 10% fetal bovine serum (Gibco, USA) and 1% penicillin-streptomycin (HyClone, USA) in a 37°C and 5% CO2 incubator. The GC cells were then treated with continuous low-dose of cisplatin in a stepwise manner to developed cisplatin resistant GC (MKN45-CDDPR) cells.

### 4.3. Cell Transfection

The PITPNA-AS1 knockdown and miR-98-5p overexpression plasmids were purchased from GeneChem (Shanghai, China). The above plasmids were delivered into MKN45 and AGS cell lines by using the Lipofectamine 3000 (Invitrogen, Carlsbad, CA, USA) reagent according to the manufacturer's instruction.

### 4.4. RNA Sequencing

The RNA sequencing process was guided and supported by GeneChem (Shanghai, China). In brief, total RNA was extracted from 3 GC patients' tissues and corresponding normal tissues by using TRIzol (Invitrogen, Carlsbad, CA). And the RNA purification was performed by using the RNA Clean XP Kit (Beckman Coulter, Kraemer Boulevard Brea, CA) and the RNase-Free DNase Set (QIAGEN, GmbH, Germany). Finally, the Illumina HiSeq 2000/2500 (Illumina Inc., San Diego, CA) was used for RNA sequencing.

### 4.5. qRT-PCR

Total RNA was extracted from GC tissues and cell lines by using Trizol reagent (Invitrogen, Carlsbad, CA, USA). The cDNA was generated by using the first-strand cDNA synthesis kit (Tiangen Biotech, Beijing, China). The expression levels of PITPNA-AS1 was tested by conducting qRT-PCR using SYBR® Premix Dimer Eraser kit (Takara Shiga, Japan). And *β*-actin was used as the inner reference. The miScript microRNA RT PCR kit (Qiagen, Toronto, ON, Canada) was used for cDNA synthesis and qRT-PCR process for miR-98-5p expression. U6 was used as the internal reference. ABI 7500 Real-Time PCR system (Applied Biosystems, Carlsbad, CA, USA) was conducted to perform the qRT-PCR process. The expression level was calculated by 2 − ΔΔCt method.

### 4.6. Western Blot

The total proteins were extracted from GC cells by using RIPA lysis buffer (Sigma-Aldrich, Darmstadt, Germany) and were quantified by BCA Protein Assay Kit (Beyotime, Shanghai, China). Then, proteins were diverted onto PVDF membranes (Millipore, USA) after separated by SDS-PAGE. The transferred PVDF membranes were blocked by using 5% skim milk and then were incubated overnight at 4°C with primary antibodies, which is including H3K27ac (1 : 1000, CST, Shanghai, China) and Histone H3 (1 : 2000, CST, Shanghai, China). Subsequently, the membranes were incubated with the secondary antibody (1 : 10000, Beyotime, Shanghai, China). Then, the enhanced chemiluminescence (ECL, Beyotime, Shanghai, China) was used to quantify the protein expression levels.

### 4.7. Cell Proliferation

In order to evaluate the proliferation and cisplatin resistance of GC cells, the cell counting kit-8 (CCK-8) kit (AbMole, USA) was used according to the manufacturer's protocol. Briefly, GC cells were seeded in 96-well plates with a density of 5 × 10^3^ cells per well, then 10 *μ*L of CCK-8 reaction solution was supplemented into each well every 24 h followed by incubation for 2 h. Then, the optical density (OD) values of GC cells at 450 nm were detected to assess cell proliferation of GC.

### 4.8. Cell Apoptosis Assay

The Caspase 3/7 Activity Apoptosis Assay Kit (Invitrogen) was used to detect the apoptosis rate of GC cells. According to the manufacturer's instruction, GC cells were plated into the 96-well plate overnight at 20000 cells per well. Then, 50 *μ*L of caspase 3/7 substrate (component A) was added into 10 mL of assay buffer (component B) to make caspase 3/7 assay loading solution. GC cells were incubated in a 37°C, 5% CO2, incubator for 6 h after treated with camptothecin to induce apoptosis. Then, GC cells were added with 100 *μ*L/well of caspase 3/7 assay loading solution, followed by supplemented with the assay loading solution at room temperature under dark conditions for 1 h of incubation. Finally, GC cells were centrifuged at 800 rpm for 2 min, then the fluorescence intensity at Ex/Em = 490/525 nm was monitored to evaluate cell apoptosis rate.

### 4.9. Dual-Luciferase Reporter Gene Assay

The plasmids of PITPNA-AS1 wild-type (PITPNA-AS1-WT) and PITPNA-AS1 mutant type (PITPNA-AS1-Mut) were cotransfected with the miR-98-5p-NC mimic into GC cells by using Lipofectamine 2000 (Invitrogen, USA). And then the miR-98-5p-WT and miR-98-5p-Mut vectors were transfected into GC cells as well. Then, dual-luciferase reporter system (Promega, Madison, WI, USA) was conducted to estimate the luciferase activities based on the manufacturer's instruction.

### 4.10. RNA Pull-Down Assay

Biotin-labeled miR-98-5p-WT and miR-98-5p-Mut were synthesized by GeneCreate (Wuhan, China) and were transfected into GC cells which were incubated with lysis buffer (Ambion, Austin, Texas, USA). Then, the GC cell lysates were incubated with the streptavidin Dynabeads (Invitgen, USA) precoated with RNase-free bovine serum albumin (BSA) and yeast tRNA (Sigma-Aldrich, USA) overnight at 4°C. After washed with washing buffer, the bound RNA was purified by using Trizol. Finally, the enrichment of PITPNA-AS1 was identified and estimated by performing qRT-PCR.

### 4.11. Statistics

The SPSS 18.0 software and the GraphPad Prism 8.2 software were used to analyze and visualize the data involved in this study. The Limma package were used for RNA sequencing analysis. A paired Student's *t*-test was used to evaluate the statistical differences between two groups. And one-way ANOVA was applied for multiple-group comparison. The Kaplan-Meier survival analysis was used to estimate the prognosis of GC patients. Each assay was independently repeated at least three times, and all the statistical results presented in this work were expressed as mean ± standard deviation (SD). A *p* value of <0.05 was indicative of statistically significant difference.

## Data Availability

Data would be made available on request by sending e-mail to the corresponding author.

## Supplementary Materials

Supplementary Materials Sup1 Figure 1: FISH assay was used to show that PITPNA-AS1 mainly resided in the plasma of MKN45 and AGS. Sup Figure 2: (A) qRT-PCR was used to detect the expression of PITPNA-AS1 in PITPNA-AS1 silence AGS cells to show the knocking down efficiency. (B) qRT-PCR was used to detect the expression of PITPNA-AS1 in PITPNA-AS1 silence MKN45 cells to show the knocking down efficiency. (C) qRT-PCR was used to detect the expression of miR-98-5p in miR-98-5p overexpression AGS cells to show the overexpression efficiency. (D) qRT-PCR was used to detect the expression of miR-98-5p in miR-98-5p overexpression MKN45 cells to show the overexpression efficiency.
