# Supplementary material for: PITPNA-AS1/miR-98-5p to Mediate the Cisplatin Resistance of Gastric Cancer
Source: J Oncol. 2022 May 7;2022:7981711. doi: 10.1155/2022/7981711 (PMC9107361; doi:10.1155/2022/7981711)
Supplement: Supplementary Materials — Sup1 Figure 1: FISH assay was used to show that PITPNA-AS1 mainly resided in the plasma of MKN45 and AGS. Sup Figure 2: (A) qRT-PCR was used to detect the expression of PITPNA-AS1 in PITPNA-AS1 silence AGS cells to show the knocking down efficiency. (B) qRT-PCR was used to detect the expression of PITPNA-AS1 in PITPNA-AS1 silence MKN45 cells to show the knocking down efficiency. (C) qRT-PCR was used to detect the expression of miR-98-5p in miR-98-5p overexpression AGS cells to show the overexpression efficiency. (D) qRT-PCR was used to detect the expression of miR-98-5p in miR-98-5p overexpression MKN45 cells to show the overexpression efficiency. [file 7981711.f1.docx]

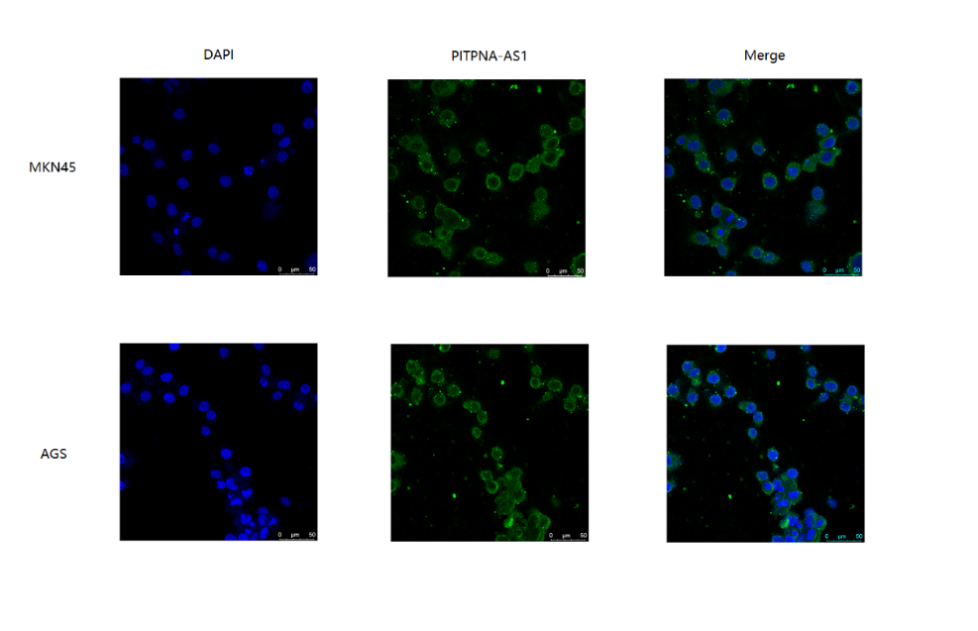


Sup1 Figure1 FISH assay was used to show that PITPNA-AS1 mainly resided in the plasma of MKN45 and AGS,.

**
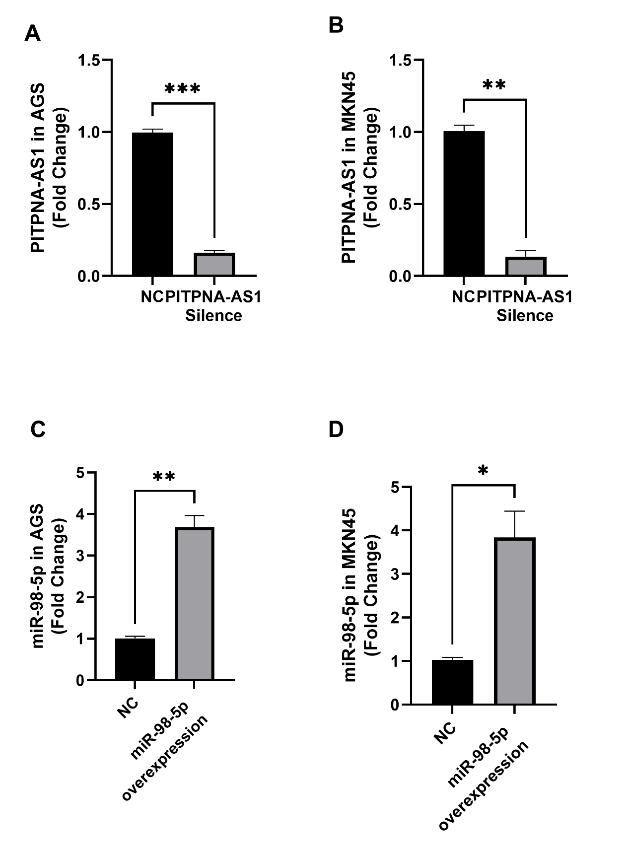
**

Sup Figure2 A qRT-PCR was used to detect the expression of PITPNA-AS1 in PITPNA-AS1 silence AGS cells to show the knocking down efficiency. B qRT-PCR was used to detect the expression of PITPNA-AS1 in PITPNA-AS1 silence MKN45 cells to show the knocking down efficiency. C qRT-PCR was used to detect the expression of miR-98-5p in miR-98-5p overexpression AGS cells to show the overexpression efficiency. D qRT-PCR was used to detect the expression of miR-98-5p in miR-98-5p overexpression MKN45 cells to show the overexpression efficiency.
